# Downregulation of EAAT-2 impairs chronic neuropathic pain via increasing of plasma glutamate after herpes zoster infection

**DOI:** 10.1038/s41598-025-01501-5

**Published:** 2025-07-01

**Authors:** Li-Na Lu, Li-Hong Mei, Xu-Shuo Li, Ji-Hong Zhang, Gao Yang

**Affiliations:** 1 Department of Dermatology, Jinshan Hospital of Fudan University, Shanghai, 201508 China; 2 Department of Clinical Laboratory, Jinshan Hospital of Fudan University, Shanghai, 201508 China

**Keywords:** Skin diseases, Infectious diseases, Predictive markers

## Abstract

Chronic neuropathic pain (CNP) is a debilitating complication of herpes zoster (HZ) with significant impact on quality of life. This study aimed to investigate the association between excitatory amino acid transporter 2 (EAAT-2) expression and plasma glutamate concentrations in HZ patients with CNP. This study was conducted with 102 consecutive patients diagnosed with HZ. Participants were divided into two groups: CNP (*n* = 51) and acute pain (ACP, *n* = 51). Pain severity was assessed using the Numerical Rating Scale. Blood samples were collected for genotype analysis, mRNA and protein extraction, and plasma glutamate measurement. EAAT-2 DNA genotyping was analyzed by polymerase chain reaction (PCR); EAAT-2 mRNA expression was analyzed by quantitative real-time PCR; EAAT-2 protein and glutamate levels were analyzed by enzyme-linked immunosorbent assay. The EAAT-2 DNA showed no significant difference in CNP and ACP patients. CNP patients exhibited lower EAAT-2 mRNA and protein levels compared to ACP patients. However, plasma glutamate levels were significantly elevated in the CNP patients. A correlation was observed between EAAT-2 protein concentration and plasma glutamate levels in the CNP group. This study demonstrates EAAT-2 mRNA downregulation, reduced EAAT-2 protein concentration, and elevated plasma glutamate levels play roles in CNP following HZ infection. These findings suggests that EAAT-2 may be a relevant target for further investigation in therapeutic development.

## Introduction

Herpes zoster (HZ) is a viral infection that predominantly affects the sensory nerves. HZ is caused by the reactivation of the varicella-zoster virus (VZV), the same virus responsible for chickenpox. After the primary infection, VZV remains dormant in the dorsal root ganglia and can reactivate later in life, which leads to HZ particularly in elderly or immunocompromised individuals^1^. One of the most distressing complications of HZ is chronic neuropathic pain (CNP). CNP is a condition that can significantly diminish the quality of life of affected individuals^2^. CNP manifests as persistent pain that continues for months or even years after the acute phase of the rash has healed. The underlying mechanisms of CNP derived from VZV are complex and not fully understood, which poses a significant challenge to effective treatment.

Glutamate is the primary excitatory neurotransmitter in the central nervous system (CNS), which is crucial for normal neuronal function, including synaptic transmission and plasticity^3^. However, excessive glutamate release or impaired clearance can lead to a pathological condition known as excitotoxicity. Under the condition of excitotoxicity, neurons are damaged and even killed with an over activation of glutamate receptors. This process has been implicated in the development and maintenance of chronic pain conditions, including chronic pain^3^. In addition, previous study showed that glutamate have potential involvement in CNP development after HZ infection^4^.

Excitatory amino acid transporter 2 (EAAT-2), also known as GLT-1, is primarily responsible for the reuptake of glutamate from the synaptic cleft in the CNS^5^. EAAT-2 plays a critical role in regulating extracellular glutamate levels, thereby protecting neurons from excitotoxic damage. Disruptions in EAAT-2 function have been associated with various neurological disorders, including epilepsy, amyotrophic lateral sclerosis, and chronic pain^6^. Previous studies showed that alterations in EAAT-2 expression or function could contribute to the pathogenesis of neuropathic pain^7,8^. The impact of EAAT-2 on glutamate homeostasis and its association with pain responses, indicating that dysregulation of EAAT-2 could lead to heightened pain sensitivity and the development of chronic pain states^9,10^. However, the role of EAAT-2 mRNA and protein levels in CNP after HZ remains largely uninvestigated.

In this study, we hypothesized that reduced EAAT-2 expression could lead to increased plasma glutamate levels, thereby contributing to the development and maintenance of CNP in HZ patients. To test this hypothesis, we investigated the association between EAAT-2 gene polymorphisms, EAAT-2 mRNA expression, EAAT-2 protein concentration and plasma glutamate levels in patients with HZ. Understanding the intricate interplay between EAAT-2 and CNP can provide valuable insights into the pathogenesis of CNP in HZ, which has the potential to the development of novel therapeutic strategies targeting glutamate signaling pathways in improving the quality of life for HZ patients suffering from debilitating CNP.

## Results

### Demographic and clinical characteristics

**Fig. 1:**
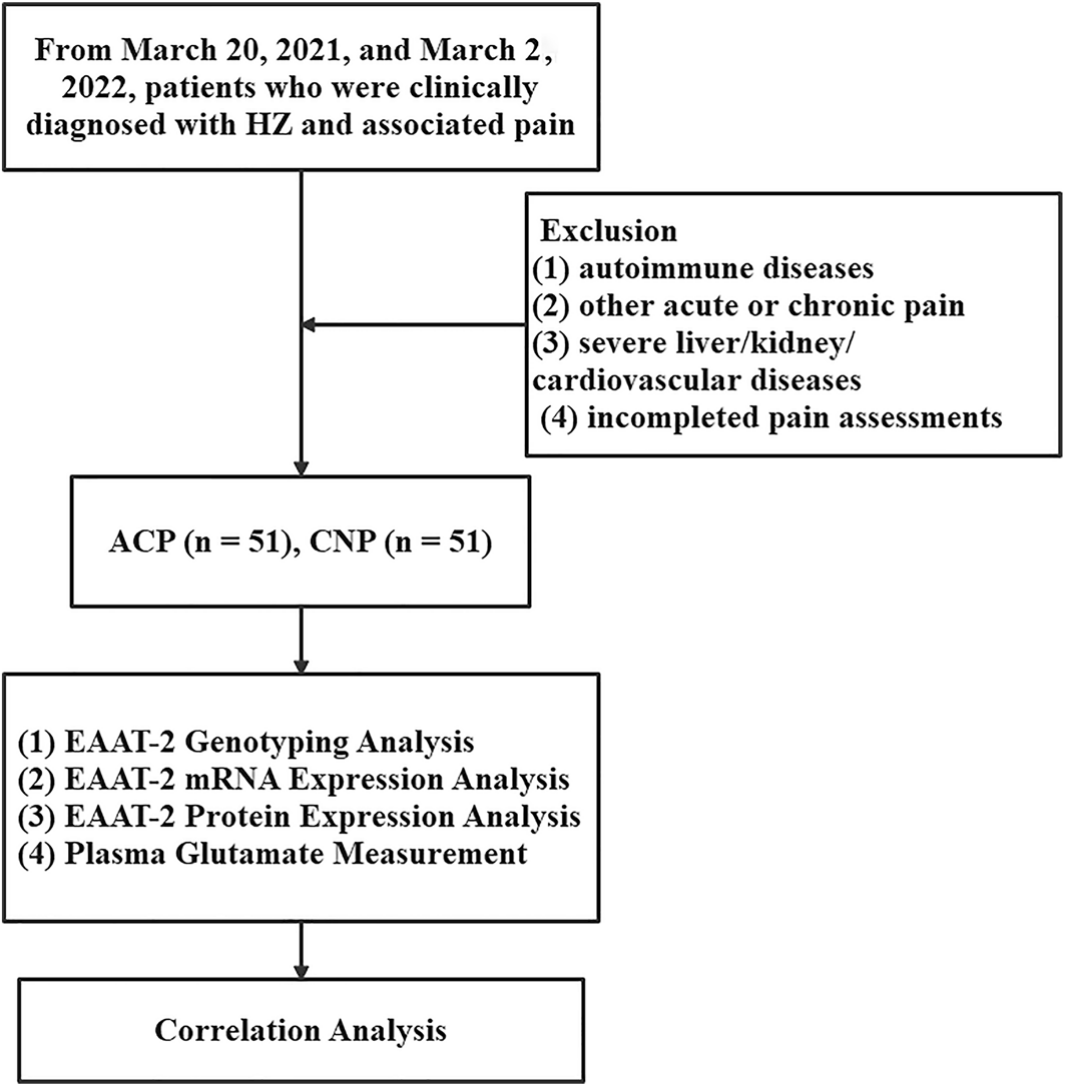
The work flow of this study.

**Fig. 2:**
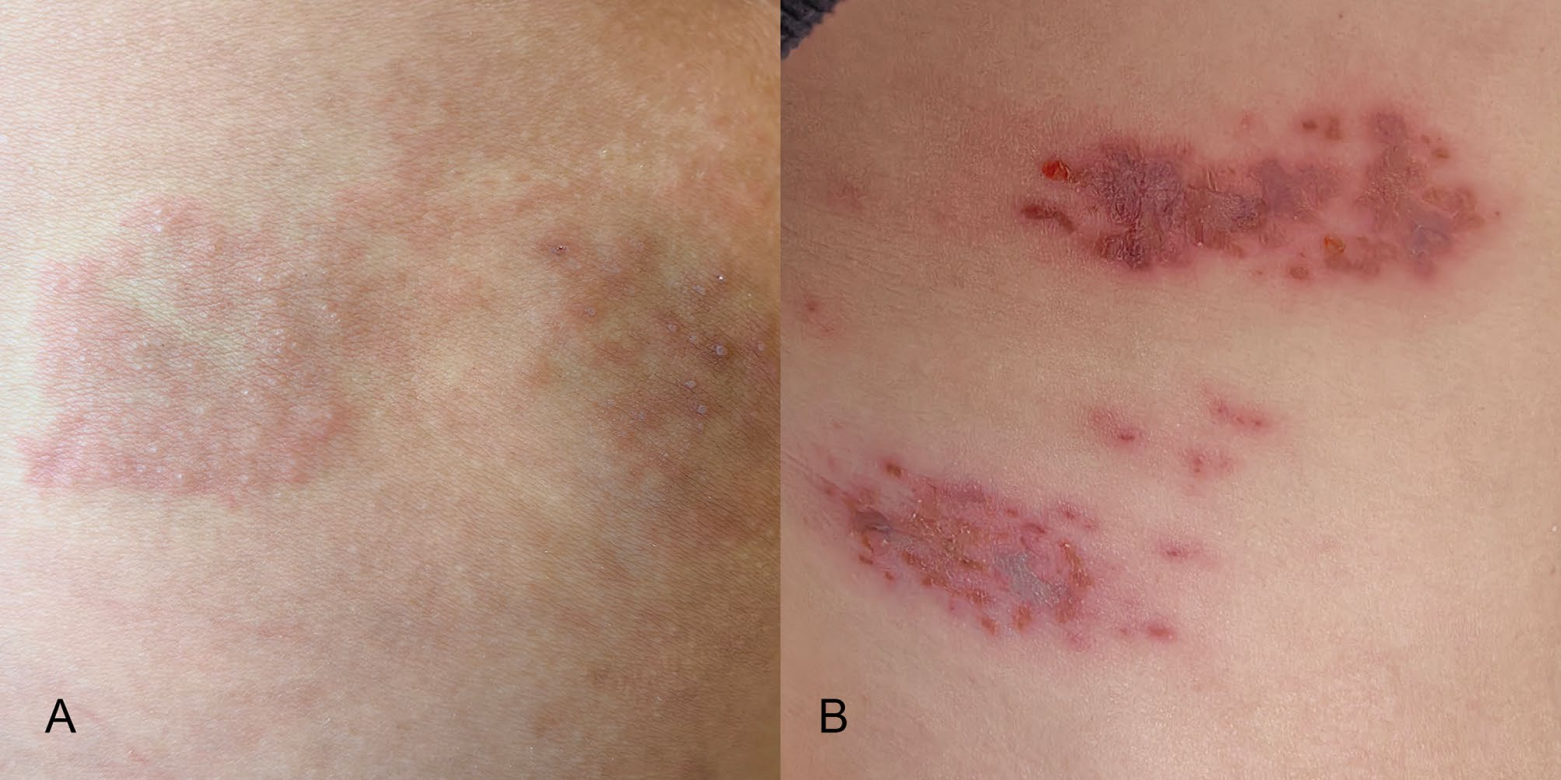
The skin lesions of two patients after herpes zoster infection. (A) The skin lesion of a patient with acute pain, which disappears after 2 weeks. (B) The skin lesion of a patient with chronic neuropathic pain, which lasts for 4 months.

The demographic and clinical characteristics of the study participants are summarized in Table 1. The mean age of the CNP group was 64 ± 13.1 years, while the ACP group had a mean age of 61 ± 11.2 years. There were no statistically significant differences in age or gender distribution between the two groups, with males comprising 52.9% of the CNP group and 43.1% of the ACP group and females comprising 47.1% of the CNP group and 56.9% of the ACP group. The work flow of this study is shown in Fig. 1. Two cases is shown in Fig. 2.

**Table 1:** Demographic and clinical characteristics in patients with chronic neuropathic pain (CNP) and acute pain (ACP) after herpes Zoster.

	ACP (*N* = 51)	CNP (*N* = 51)	*p*
Gender			
Female	29 (56.9%)	24 (47.1%)	0.428
Male	22 (43.1%)	27 (52.9%)	
Age (year)	61 ± 11.2	64.7 ± 13.1	0.186
Sites			
Abdomen	7 (13.7%)	3 (5.9%)	0.417
Abdomen and back	3 (5.9%)	9 (17.6%)	
Back	9 (17.6%)	10 (19.6%)	
Chest	2 (3.9%)	3 (5.9%)	
Chest and back	18 (35.3%)	13 (25.5%)	
Head	9 (17.6%)	11 (21.6%)	
Limb	3 (5.9%)	2 (3.9%)	
Duration (month)	1.7 ± 0.5	6.6 ± 4.0	< 0.001
Peak time (week)	1.4 ± 0.6	2.0 ± 0.7	< 0.001
Area			
Large	8 (15.7%)	10 (19.6%)	0.437
Mid	24 (47.1%)	28 (54.9%)	
Small	19 (37.3%)	13 (25.5%)	

Pain characteristics varied significantly between the two groups. The CNP group exhibited a significantly longer duration of pain compared to the ACP group. Additionally, the peak time of pain was delayed in the CNP group compared to the ACP group (Table 1). The most common sites of pain in the CNP group were the chest and back (25.5%), followed by the head (21.6%) and back (19.6%). The most common sites of pain in the ACP group were the chest and back (35.3%), followed by the head (17.6%) and back (17.6%).

### EAAT-2 genotyping analysis

**Fig. 3:**
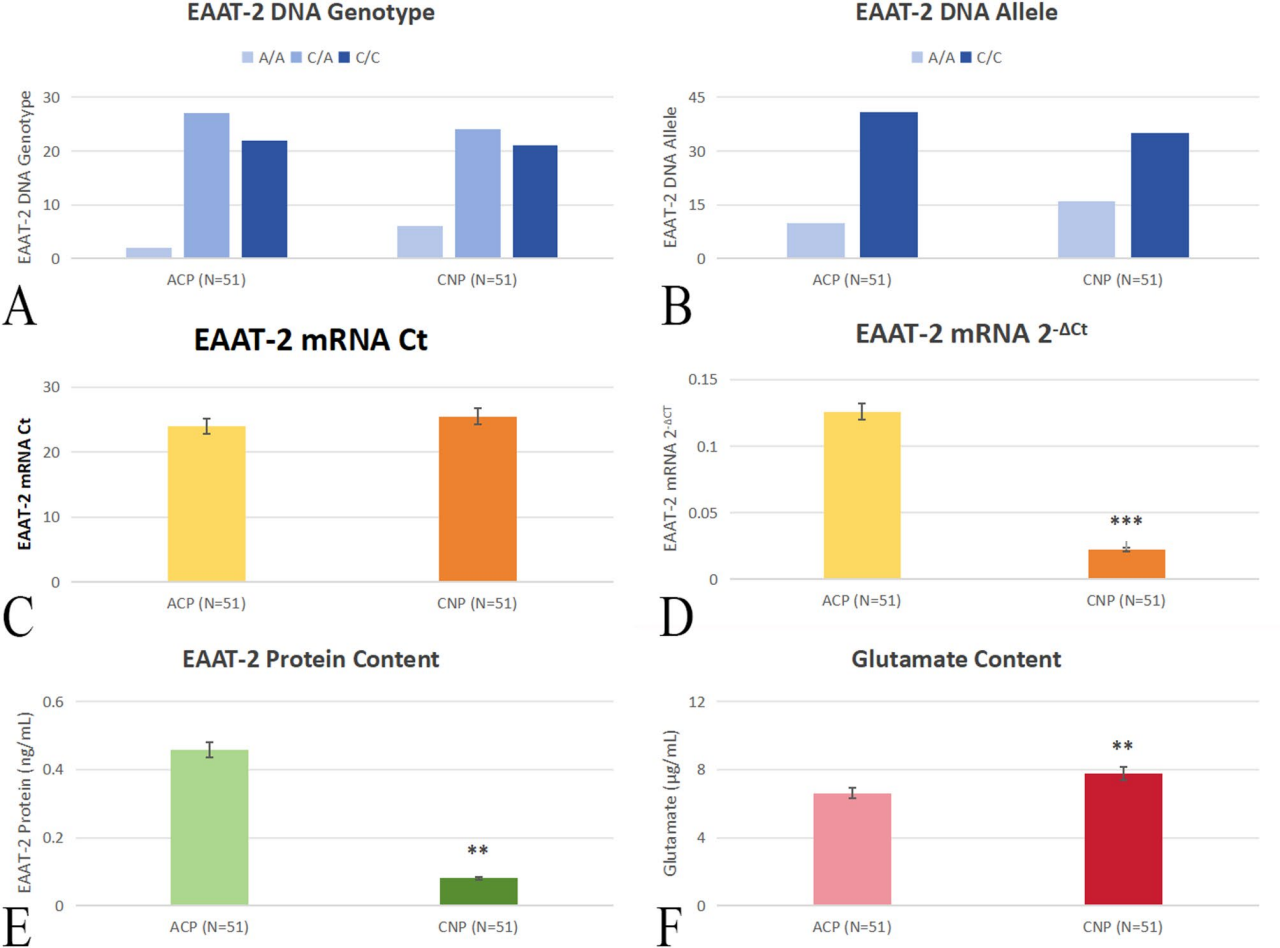
EAAT-2 genetic and molecular characteristics in ACP and CNP groups. (A) EAAT-2 genotype distribution bar graph showing the frequency of different EAAT-2 genotypes (A/A, A/C, C/C) in ACP and CNP groups. (B) Lower EAAT-2 mRNA expression (2^−ΔCt^) is showed in CNP group. (C) Lower EAAT-2 protein concentration is shown in CNP group. (D) Higher glutamate levels is shown in CNP group. *, *p* < 0.05; **, *p* < 0.01; ***, *p* < 0.001; A/A, two identical “A” alleles; C/A, one “C” allele and one “A” allele; C/C, two identical “C” alleles.

The analysis of EAAT-2 gene polymorphisms revealed no significant differences in the distribution of genotypes or alleles between the CNP and ACP groups (Fig. 3). The frequencies of the C/C genotype were 41.2% and 43.1% in CNP and ACP groups, while the C/A genotype was observed in 47.1% of the CNP group and 52.9% of the ACP group. The A/A genotype was relatively rare, occurring in 11.8% of the CNP group and 3.9% of the ACP group. The allele frequencies also did not differ significantly between the groups, with the C allele being more prevalent (68.6% in CNP vs. 80.4% in ACP).

### EAAT-2 mRNA expression analysis

Quantitative real-time PCR analysis demonstrated a significant downregulation of EAAT-2 mRNA expression in the CNP group compared to the ACP group. The EAAT-2 mRNA expression was normalized to GAPDH. The 2^−ΔC^t value of the EAAT-2 mRNA was 0.02 ± 0.01 in the CNP group, which is lower than that of 0.12 ± 0.08 in the ACP group (Fig. 3).

### EAAT-2 protein expression analysis

The analysis of plasma EAAT-2 protein levels further supported the findings from the mRNA expression analysis. A significant reduction in EAAT-2 protein concentration was observed in the CNP group compared to the ACP group. The EAAT-2 protein concentration was 0.08 ± 0.06 in the CNP group, significantly lower than the 0.45 ± 0.62 observed in the ACP group (Fig. 3).

### Plasma glutamate levels analysis

Plasma glutamate levels were significantly elevated in the CNP group compared to the ACP group. The plasma glutamate concentration was 7.7 ± 2.13 in the CNP group, compared to 6.6 ± 1.33 in the ACP group (Fig. 3).

### Correlation analysis

Correlation analysis was performed to assess the relationships between EAAT-2 protein and plasma glutamate levels in both groups. In the CNP group, a significant negative correlation was found between EAAT-2 protein and plasma glutamate levels, indicating that lower EAAT-2 protein levels are associated with higher plasma glutamate concentrations. This correlation was not observed in the ACP group. A subgroup analysis was conducted to explore the potential impact of age and gender on the observed associations between EAAT-2 expression, glutamate levels, and pain outcomes. The results showed that the observed associations were consistent across different age groups and between males and females.

## Discussion

This study showed that the downregulation of EAAT-2 mRNA expression and protein concentration, coupled with elevated plasma glutamate levels, played a significant role in the development and maintenance of CNP following HZ infection. These findings contribute to our understanding of the molecular mechanisms underlying CNP and suggest potential therapeutic targets for managing this debilitating condition.

EAAT-2 is a key regulator of extracellular glutamate levels in the CNS, responsible for clearing glutamate from the synaptic cleft and preventing excitotoxicity. When EAAT-2 expression is reduced, glutamate accumulates in the synaptic space and potentially leads to neuronal damage and increased pain sensitivity. In this study, the genotype frequencies of EAAT-2 polymorphisms did not differ between CNP and ACP patients after HZ infection. A previous study showed that no difference in the genotype frequencies of EAAT-2 polymorphisms in patients with chronic daily headache^11^. However, a higher frequency of analgesic usage was found inpatients with the A allele compared to those without it. This result implies a potential genetic influence on the progression of transition into chronic headache^11^. The results of this study suggests that EEAT-2 polymorphism does not contribute to the transition from ACP to CNP after HZ infection, although a prevalent of C allele was observed. Previous study shows that alterations in EAAT-2 mRNA expression can exacerbate pain by failing to control excessive glutamate levels effectively^12^. Modulating EAAT-2 activity could help restore glutamate balance and alleviate chronic pain^13^. The observed downregulation of EAAT-2 mRNA expression and protein concentration in the CNP group suggests that this result is likely due to post-transcriptional or environmental factors rather than genetic predisposition.

Glutamate is the primary excitatory neurotransmitter in the CNS and plays a crucial role in synaptic transmission and plasticity. Elevated plasma glutamate levels observed in the CNP group further emphasize the role of glutamate in pain pathophysiology^14,15^. However, when glutamate is not adequately cleared from the synaptic cleft, it can lead to excitotoxicity. This process is closely linked to chronic pain states^2^. The significant correlation between reduced EAAT-2 protein concentrations and elevated plasma glutamate levels in the CNP group suggests a mechanistic link between EAAT-2 downregulation and glutamate-mediated excitotoxicity. This finding supports the hypothesis that impaired glutamate clearance contributes to the transition from acute to chronic pain^16,17^. This also aligns with existing literature, which has shown that increased glutamate levels are associated with various forms of chronic pain, including neuropathic pain^18^. Impairs glutamate clearance also leads to NMDA receptor overactivation and neuronal hyperexcitability-key factors in central sensitization^19^. Furthermore, other mechanisms may contribute to CNP, including glial cell activation, oxidative stress and inflammatory cytokines like TNF-α and IL-1β^20^.

Pharmacological agents that enhance EAAT-2 expression or function could potentially reduce glutamate levels and prevent excitotoxic damage, thereby alleviating pain. For example, β-lactam antibiotics like ceftriaxone have been shown to upregulate EAAT-2 expression and reduce pain in animal models of neuropathic pain^5^. Given the findings of this study, targeting EAAT-2 and glutamate signaling pathways presents a promising therapeutic approach for CNP. This highlights the importance of timely therapeutic interventions inpatients with HZ to prevent the development of CNP. Pharmacological strategies, such as small-molecule EAAT-2 activators and gene therapy approaches could be considered explored to restore glutamate balance. Furthermore, combining EAAT-2 modulation with anti-inflammatory or neuroprotective therapies may offer a more effective strategy for managing CNP. Future research should focus on the development and clinical testing of EAAT-2 modulators to assess their efficacy in preventing or treating CNP.

While this study provided valuable insights into the association between EAAT-2, glutamate and CNP, several limitations should be acknowledged. First, the relatively small sample size may limit the generalizability of the findings. Larger cohort studies for external validation with more diverse populations should be carried out. Second, the study’s cross-sectional design restricts the ability to interpret the causal relationship between EAAT-2, glutamate, and CNP. Longitudinal studies are needed to track EAAT-2 and glutamate changes over time and determine their role in the transition from acute to chronic pain. Future research should explore whether interventions targeting EAAT-2 expression can prevent or mitigate the development of CNP in HZ patients. Finally, further research is needed to investigate other polymorphisms, epigenetic modifications, or regulatory mechanisms affecting EAAT-2 expression, which may provide deeper insights into individual variability in pain susceptibility and response to treatment.

In conclusion, our findings demonstrate an association between EAAT-2 mRNA downregulation, reduced protein concentration, and elevated plasma glutamate levels in CNP following HZ infection. The mechanisms link between EAAT-2 regulation and CNP remains to be fully elucidated. Future studies incorporating pathway inhibition and additional internal controls are necessary to confirm the involvement of EAAT-2 in CNP pathogenesis. Our study suggests that EAAT-2 may be a relevant target for further investigation in therapeutic development.

## Methods

### Ethical statement

This study was conducted in accordance with the principles outlined in the Declaration of Helsinki and was approved by the Institutional Review Board of Jinshan Hospital of Fudan University (Approval No. JIEC2021S18). Written informed consent was obtained from all participants prior to their inclusion in the study. The ethical considerations were meticulously adhered to, ensuring the rights, safety, and well-being of all participants were protected throughout the research process.

### Study design and participants

This study employed a case-control design to investigate the association between the rs4354668 polymorphisms in the EAAT-2 gene, EAAT-2 mRNA expression, EAAT-2 protein concentration, and plasma glutamate levels in patients with CNP following HZ infection. The study was conducted between March 20, 2021, and March 2, 2022, and involved 102 consecutive patients who were clinically diagnosed with HZ and associated pain.

Participants were recruited from the dermatology department and were divided into two groups: the CNP group (*n* = 51), which included patients who had experienced CNP for at least three months, and the acute pain (ACP) group (*n* = 51), which included patients who had experienced acute pain for less than three months following the onset of HZ. The inclusion criteria for both groups were: (1) a clinical diagnosis of HZ according to the International Classification of Diseases, 10th Revision (ICD-10), and (2) the ability to undergo a numerical rating scale (NRS) pain assessment. Exclusion criteria included: (1) the presence of concurrent autoimmune diseases, (2) concurrent tumors or other acute or chronic pain conditions, (3) severe liver, kidney, or cardiovascular diseases, and (4) inability to complete pain assessments.

### Pain assessment

Pain severity was assessed using the NRS, a validated tool for quantifying pain intensity. The NRS scores range from 0 to 10, with 0 representing no pain and 10 representing the worst pain imaginable. Pain characteristics were documented for all participants (including continuous and/or episodic burning, stabbing, or electric shock-like pain, touch-induced pain, and hypersensitivity). The duration and peak time of pain were also recorded, with the duration of pain defined as the time elapsed since the onset of pain, and peak time defined as the time taken to reach maximum pain intensity.

### Blood sample collection and processing

Fasting venous blood samples (4 mL) were collected from each patient in the morning. The blood was drawn into anticoagulant tubes and immediately stored at – 80 °C until further analysis. Peripheral blood mononuclear cells (PBMCs) were isolated from the collected blood samples for subsequent DNA and mRNA analyses. Plasma was used for protein and glutamate analyses. All blood samples were collected within 7 days of the onset of HZ.

### EAAT-2 genotyping analysis

DNA was extracted from PBMCs using the standard phenol-chloroform extraction method. Polymerase chain reaction (PCR) was performed to amplify the rs4354668 polymorphism region of the EAAT-2 gene. The following primers were used: forward primer 5’-CTGCCACCTGTGCTTTGCT-3’ and reverse primer 5’-GAGGGATCGCCTGCAAATCC-3’. The amplified DNA fragments were subjected to restriction fragment length polymorphism analysis to identify EAAT-2 genotypes. The PCR products were digested with the appropriate restriction enzyme, and the resulting fragments were separated by gel electrophoresis to determine the genotype.

### EAAT-2 mRNA expression analysis

Total RNA was extracted from PBMCs using TRIzol reagent according to the manufacturer’s instructions. The quality and concentration of the extracted RNA were assessed using a spectrophotometer. Reverse transcription of the RNA into complementary DNA (cDNA) was performed using a commercially available reverse transcription kit. Quantitative real-time PCR (qPCR) was then conducted to measure EAAT-2 mRNA levels using specific primers designed for EAAT-2. GAPDH was used as the reference gene to normalize the expression levels. The relative expression of EAAT-2 mRNA was calculated using the 2^−ΔCt^ method. The threshold cycle (Ct) values of EAAT-2 and GAPDH were first recorded. Then the difference between EAAT-2 and GAPDH Ct values was calculated as ΔCt. Last, 2^−ΔCt^ was calculated to represent the mRNA levels of EAAT-2.

### EAAT-2 protein expression analysis

To assess EAAT-2 protein levels, plasma was separated from blood samples by centrifugation at 3,000 rpm for 10 min. The plasma EAAT-2 protein levels were quantified using a commercially available enzyme-linked immunosorbent assay (ELISA) kit (manufacturer: Abnova, catalog number: abx494583), following the manufacturer’s protocol. Briefly, plasma samples were added to wells coated with an anti-EAAT-2 antibody, followed by the addition of a secondary antibody conjugated to an enzyme. After the addition of a substrate solution, the enzyme reaction produced a colorimetric signal proportional to the amount of EAAT-2 protein present in the sample. The absorbance was measured at 450 nm using a microplate reader, and EAAT-2 protein concentrations were calculated based on a standard curve.

### Plasma glutamate measurement

Plasma glutamate levels were measured using an ELISA kit (manufacturer: Abbexa, catalog number: KA1909) specifically designed for the quantification of glutamate. Plasma samples processing and ELISA procedure were performed in the similar manner as for the EAAT-2 protein analysis.

### Statistical analysis

Statistical analyses were performed using R software version 4.3.0. Descriptive statistics, including mean ± standard deviation, and frequency distributions, were used to summarize the demographic and clinical characteristics of the study participants. After normal distribution test, independent-samples t-tests or Mann-Whitney U test were used to compare continuous variables between the CNP and ACP groups. Chi-square tests were employed to assess differences in categorical variables. Pearson’s correlation coefficient was used to evaluate the relationships between EAAT-2 mRNA expression and protein concentration and plasma glutamate level. Statistical significance was set at *p* < 0.05.

A power analysis was conducted to ensure that the sample size was adequate to detect significant differences between the groups with sufficient statistical power. The analysis indicated that a sample size of 25 per group was sufficient to detect a moderate effect size with a power of 0.80 at an alpha level of 0.05.

## Data Availability

The raw data supporting the conclusions of this article will be made available by the authors (Gao Yang, dr_yanggao@163.com), without undue reservation. The raw data can be also found in the GenBase in National Genomics Data Center, Beijing Institute of Genomics, Chinese Academy of Sciences/China National Center for Bioinformation, under accession number 2025BAT00714 that is publicly accessible at https://ngdc.cncb.ac.cn/genbase.
